# Assessing the impact of Medical Education's Innovation & Entrepreneurship Program in China

**DOI:** 10.1186/s12909-024-05467-2

**Published:** 2024-05-10

**Authors:** Xiandi You, Wenyi Wu

**Affiliations:** 1 Xiangya Hospital, Xiangya School of Medicine, Central South University, Changsha, Hunan China; 2 Department of Ophthalmology, Xiangya Hospital, Central South University, Changsha, China; 3 Hunan Key Laboratory of Ophthalmology, Changsha, China; 4 National Clinical Research Center for Geriatric Disorders, Xiangya Hospital, Central South University, Changsha, China

**Keywords:** I&E program, Medical education, Questionnaire, Research abilities, Entrepreneurial abilities

## Abstract

**Objective:**

A growing number of clinical undergraduates are chosen to enter institutions for higher education biotechnology and industry workforce, though most need more laboratory experience training and business practice. Innovation and Entrepreneurship Program (I&E Program) can benefit from biological experiment and commercialization training largely absent from standard clinical medical educational curricula. Our study investigates the impact and status of the I&E Program in enhancing medical students’ research and entrepreneurial abilities and provides recommendations for improving this program.

**Methods:**

A cross-sectional study was applied by delivering a questionnaire to survey medical students from Central South University who participated in the I&E Program. The questionnaire consisted of three parts: basic information, the impact of the I&E Program on medical students’ research and entrepreneurial abilities, and attitudes and recommendations regarding the I&E Program.

**Results:**

Many students participating in the I&E Program have received competition awards and improved their academic experience, article writing, and application patents. Their research-related abilities have been enhanced, including in-lab techniques, theoretical research skills, data analysis knowledge, clinical research skills, experimental research skills, entrepreneurship, data analysis ability, teamwork, and communication. While 73.93% of students express satisfaction with the I&E Program, there are still several areas of improvement, including more robust practical components, increased support, and enhanced teamwork.

**Conclusion:**

The scale of the I&E Program is rapidly expanding to address scientific research or business skills needed by college students in the new era. However, more programs still need to be discontinued during their further study. The I&E Program significantly enhances research abilities and fosters confidence in their study. This analysis emphasizes the importance of research-oriented and interdisciplinary education for students’ holistic development in medical schools compared with formal medical education.

## Introduction

The National Innovation and Entrepreneurship Training Program for College Students (I&E Program) in China originated from the Ministry of Education’s policy recommendations in 2010. These recommendations aimed to promote innovation and entrepreneurship education in higher education, fostering students’ practical skills and innovative spirit. Implemented in 2011, the program received significant participation, with 16,300 projects submitted by Central South University under the ministries for approval [1]. More universities joined the program, which expanded nationwide with local government support and active university involvement, garnering widespread participation. In 2021, Hunan Province of China approved 11,394 I&E Program with a budget of 87.15 million yuan, involving 49,451 student participants. Central South University initiated 1,731 programs, including 432 in medical disciplines, comprising 391 innovative programs and 41 entrepreneurial programs [2, 3]. American allopathic medical schools typically accept between 2 and 36% of their class size into I&E Program [4], whereas Chinese medical schools show broader participation. At least 2,100 undergraduate medical students are participating in the I&E Program in 2023 at Central South University, and this number is projected to grow. A survey at Yangtze University found 61.4% of clinical medicine majors involved in the Program [5], with similar expectations at Xiangya Medical School of Central South University.

The I&E Program provides a platform to develop their innovative ideas into lab experience or viable business. It aims to cultivate high-level innovative talents who can meet the needs of building an innovative-oriented nation [6]. It empowers students to conduct independent research, program design, implementation, and analysis under instructor guidance. They receive mentorship from seasoned scientists and industry experts. What’s more, the programs provide funding, access to cutting-edge facilities, and support for intellectual property protection. They encourage student collaboration and networking, fostering team formation and joint work. Universities annually solicit program proposals from students, securing financial backing at national, provincial, or university levels through application and evaluation processes. The programs fall into three categories: innovation training, entrepreneurship training, and entrepreneurship practice. They usually span a year, with universities conducting periodic inspections involving reports and poster presentations.

Interest in I&E education in healthcare is growing. However, formal educational programs in this field are relatively rare, and there needs to be more knowledge about the status and practices of I&E education in healthcare. To address these unmet needs, medical students inclined towards I&E can pursue these interests through graduate degrees, innovation scholarships, and other courses [4, 7]. Also, it is necessary to continually report innovations in health education literature to identify successes and challenges in medical I&E education [8]. Existing research rarely includes nationwide I&E programs in Chinese healthcare [9]. The impetus for our study stemmed from a multifaceted understanding of clinical undergraduate education’s current landscape and evolving demands. Moreover, we want to assess current educational programs and recommend program improvement. Beyond evaluating the current status, there was a clear need to identify areas of improvement for the I&E Program to serve its participants better. Given the rapid expansion of such programs, it was imperative to provide evidence-based recommendations to enhance their effectiveness in meeting the new era’s demands on college students. We want to enhance the research and entrepreneurial abilities of medical students and improve their implementation and talent cultivation at medical schools.

In preparation for further survey, we first sought to identify the core I&E competencies we expect from all undergraduate participants and identify some concepts. The Entrepreneurship Competence Framework (EntreComp) generally defines entrepreneurship competency as the ability to turn ideas into actions [8]. Merriam-Webster dictionary defines “innovation” as a new idea, method, or device, which is core to bringing new approaches into a field. In business literature, innovation and entrepreneurship are widely regarded as closely intertwined concepts [10]. No medical I&E programs have publicly proposed a formal competency for the innovation [4]. However, EntreComp identified 15 competencies that would generate an “entrepreneurial mindset” for all citizens [8]. Another group validated all 15 competencies as curricular competencies in I&E for biomedical research trainees [11].

We also want to investigate what research competencies participants can gain from the I&E Program because research skills are fundamental for future clinician-researchers [12]. Some core competencies for clinical and translational scientists were also recognized [13]. Kansas City University defines six significant areas where students could demonstrate research competency [14]: project understanding, technical skills, attention to detail, analytical ability, communication with preceptor and research team members, and professionalism. These research competencies align with the Accreditation Council for Graduate Medical Education (ACGME) core competencies of Medical Knowledge. “Developing research skills in medical students: AMEE Guide No. 69,” published by The International Association for Health Professions Education (AMEE), defined several skills for medical students wanting to develop research skills. It isn’t easy to establish explicit connections between specific skills and innovation. The extensive skills identified in the literature as fostering innovation include fundamental abilities such as reading, academic skills, and technical expertise, as well as “soft” skills like problem-solving, teamwork ability, and leadership [12]. Competency is a cluster of related knowledge, attitudes, and skills that affect a significant part of one’s job [15]. Generally, the research competencies we hope to discuss include hard skills, most identified in AMEE Guide No. 69, and soft skills, aligned with I&E competencies.

## Methods

### Research design

This study adopts a cross-sectional survey design to understand the current levels of research competence among undergraduate medical students who have participated in the I&E Program. We employed a stratified random sampling method. This approach involved dividing the population into strata based on characteristics such as year of study, specialization, and prior exposure to research and entrepreneurial activities. The primary research questions include:

1. Does the I&E Program positively impact the I&E competencies and research competencies of medical students?
2. What challenges and obstacles do medical students face in the I&E Program?
3. Are medical students satisfied with the I&E Program, and how can it be improved?

### Questionnaire design

To assess medical students’ research competency, a total of 66 closed-ended items based on the assessment form by Kansas City University [16], CRAI-12 [17], and AMEE Guide No. 69 [18] were employed to explore perceptions of their familiarity or mastery of knowledge related to the I&E Program and various their confidence in multiple competencies. These items were divided into the following sections:

1. General Student Information (10 items): This section covered demographics such as age, gender, and academic year.
2. Research Skills (3 items): This section included questions related to laboratory techniques, theoretical research skills, and knowledge of data analysis.
3. Research Competencies (by Different Domains):
  Experimental Research Competencies (12 items)
  Clinical Research Competencies (10 items)
  Entrepreneurial Abilities (6 items)
4. Data Analysis Ability (4 items): This section assessed students' data processing and analysis proficiency.
5. Communication Skills (7 items): This section evaluated written and oral communication skills.
6. Satisfaction Levels and Advice (14 items): This section gauged students' satisfaction with the Innovation and Entrepreneurship program and collected their suggestions for improvement.

In the part of the study rooted in the “Self-Efficacy Theory [19]” and the “Social Cognitive Career Theory [20]”, we employed a Likert scale. This scale prompted students to rate their perspectives or experiences on a 5-point scale, ranging from “Strongly Disagree” to “Disagree,” “Neutral,” “Agree,” and “Strongly Agree.” An open-ended question was also included to elicit students’ feedback or opinions regarding the Innovation and Entrepreneurship program.

It is important to note that in the section concerning Research Competencies, each student was required to complete one subsection out of Clinical Research Skills, Experimental Research Skills, or Entrepreneurial Abilities based on the direction of their program participation.

These details in the questionnaire design will facilitate a comprehensive understanding of students’ research abilities and satisfaction levels across various dimensions and how their perspectives and experiences correlate with self-efficacy and social cognitive career theory.

### Participants

After conducting a small-scale distribution, the questionnaire design was re-evaluated, and the time required for typical responses was estimated. Following the completion of questionnaire collection, researchers removed invalid questionnaires based on the time respondents took to answer questions and the completeness of questionnaire responses.

A total of 200 undergraduates were randomly selected.  An online survey using an internet platform was conducted, and 188 valid responses were collected. These survey responses will be utilized to address research questions and conduct relevant statistical analyses. Participants were informed about data usage and confidentiality before participating in the survey, and their responses were anonymous.

The sample was sourced from the Xiangya School of Medicine (Central South University) students, encompassing individuals from various academic disciplines in medicine, year levels, and backgrounds. Participants were recruited by sharing the survey link on the university's online platforms. The recruitment process was extended over a specific duration to ensure diverse responses.

## Results

### Program characteristics

Out of the 188 surveyed students, the predominant group comprises third-year clinical medicine students. They are in their third year of clinical medicine. Clinical medicine students have the highest number of students in most schools and are most interested in participating in scientific research during their third year. 61.70% of students have served as a leader at least once; among them, 67.53% have received funding at least at the provincial level (Table 1).

**Table 1:** Participant demographics and program involvement characteristics

Characteristics	Group	Number	Percentage
Gender	Male	94	50%
	Female	94	50%
Grade	First year	8	4.26%
	Second year	37	19.68%
	Third year	96	51.06%
	Fourth year	34	18.09%
	Fifth year	9	4.79%
	Graduate	4	2.13%
Major	Basic Medical Sciences	17	9.04%
	Clinical Medicine	98	52.13%
	Stomatology	17	9.04%
	Public Health and Preventive Medicine	20	10.64%
	Traditional Medicine	1	0.53%
	Forensic Medicine	3	1.60%
	Nursing	2	1.06%
	Pharmacy	14	7.45%
	Biological Sciences	13	6.91%
	Medical Laboratory Technology	3	1.60%
Age	under 18	1	0.53%
	18	2	1.06%
	19	16	8.51%
	20	55	29.26%
	21	61	32.45%
	22	41	21.81%
	above 23	12	6.38
Number of I&E Program participanted in	1	68	36.17%
	2	86	45.74%
	3	32	17.02%
	4	2	1.06%
	5	0	0.00%
Participate in the program as	Leader^a^	72	38.30%
	Participant	116	61.70%
The highest level of program as leader	School level	21	29.17%
	Provincial level	32	44.44%
	National level	19	26.39%
The highest level of program as participant	School level	33	28.45%
	Provincial level	61	52.59%
	National level	22	18.97%

^a^If a participant has been involved in two or more IE Program and has been as a leader at least once, they are classified as leader

### Research skills

#### Lab techniques

**Fig. 1:**
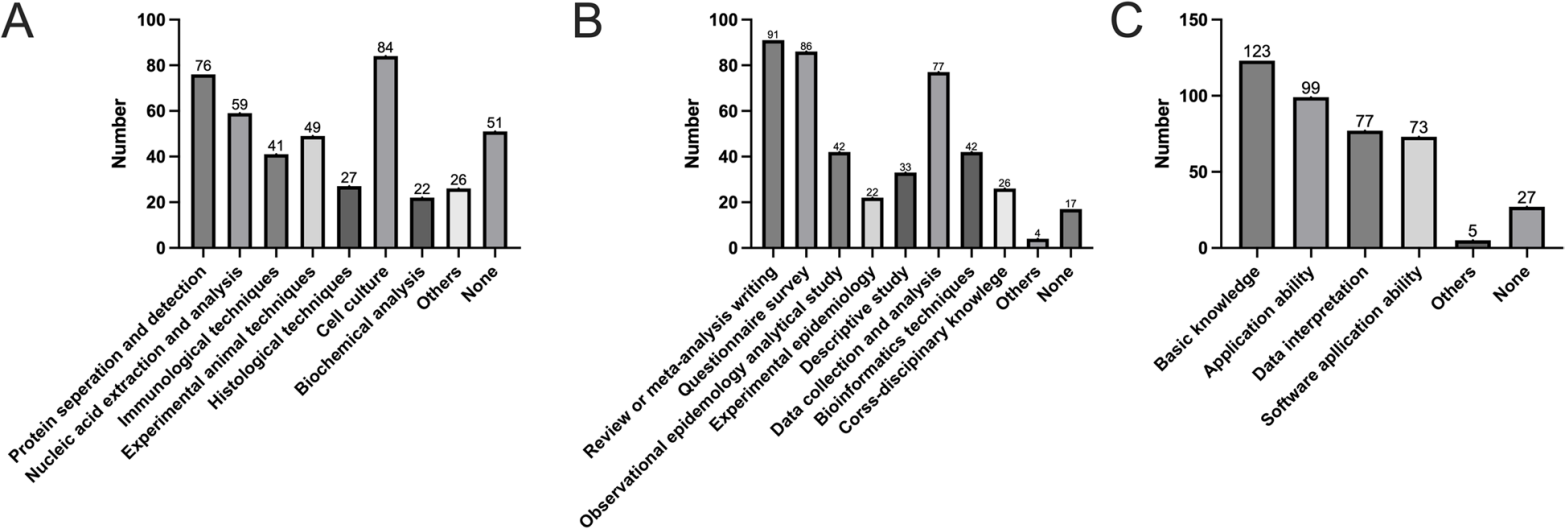
**A **Learning Outcomes in Lab Techniques from the I&E Program. **B** Learning Outcomes in Theoretic Research Skills from the I&E Program. **C** Learning Outcomes in Data Analysis Knowledge from the I&E Program

Competence in research means possessing several skills and abilities to perform specific research. According to the survey in this study, many students became familiar with laboratory techniques. The data are shown in Fig. 1A. Among the participants of the innovation program, the top three standard laboratory techniques are cell culture, protein separation and detection, and Nucleic acid extraction and analysis. Such results may be influenced by their versatility, applicability across different research areas, and ability to provide valuable insights into cellular and molecular processes.

Although most students have mastered some laboratory techniques, there still needs to be a higher level of proficiency in other techniques and methods among students, such as immunological, experimental animal models, histological techniques, and biochemical analysis. However, 51 students (27.13%) claimed they didn’t learn any related methods.

#### Theoretic research skills

Theoretical research skills are vital for medical students, fostering critical thinking, research aptitude, and lifelong learning. Through the I&E Program, many students have become familiar with or mastered research methods and techniques. The data are shown in Fig. 1B. Systematic review and meta-analysis writing (48.40%) and questionnaire surveys (45.74%) are the most common research methods. These methods play an essential role in scientific research by collecting and organizing research data and understanding people’s perspectives and opinions.

Data collection and analysis are also essential skills that many students have acquired (40.95%). Statistical methods are crucial in research, helping students collect and analyze data to draw scientific conclusions.

Various epidemiological methods are also techniques that some students have mastered. These methods are commonly used in epidemiological research to understand and analyze the spread and impact of diseases.

22.34% of these students have also acquired skills in bioinformatics. Bioinformatics is an interdisciplinary field that combines computer science and biology, and it can be used to analyze and interpret biological data. Because experimental epidemiological methods often involve research processes and are closely integrated with clinical practice, they may not be suitable for undergraduate innovation programs.

Only 26 (13.82%) students have acquired skills in other disciplines, such as medical engineering and cross-disciplinary knowledge. These skills may be related to the specific programs or research directions they are involved in. It cannot be denied that only a few students have been exposed to cross-disciplinary knowledge in medicine. This is because there is a need for more professionals in related fields, and students need a greater understanding of this field.

#### Data analysis knowledge

Through the innovation program, students are familiar with or have mastered data analysis in life science. 65.42% of the students are familiar with or have mastered fundamental concepts of statistics (Fig. 1C), commonly used statistical methods, and analysis steps. This is important for their ability to analyze and interpret results effectively.

Regarding application ability, over half of the students select appropriate statistical methods and analyze data based on the situation. This indicates they have some data analysis skills and can apply statistical methods to real-world problems. In data interpretation, 40.95% of the students can explain the meaning and impact of statistical results (Fig. 1C). This is crucial for them to draw meaningful conclusions and inferences from the data. Surprisingly, 38.83% of the students can use statistical software such as SPSS, R, Python, etc. for data analysis. These software tools are widely used in modern scientific research, and students have a relatively high level of proficiency in using them.

### Research competencies

**Fig. 2:**
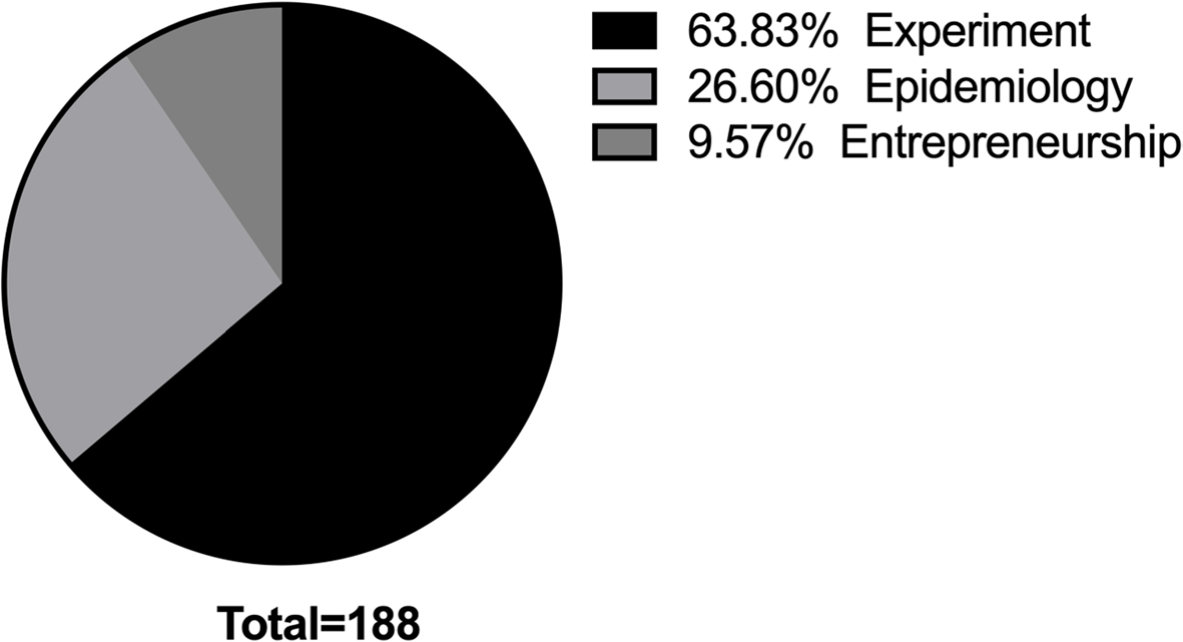
Distribution of Student Research Programs by Type

According to the research in which students participate, they can be divided into experimental, epidemiological, and entrepreneurial programs, and students’ research capabilities can be investigated accordingly. The I&E Program type distribution is shown in Fig. 2.

The curriculum and mentorship focus often influence the research programs medical students participate in. Considering schools and mentors prioritize experimental research, medical students are more likely to be involved in such programs. Meanwhile, Medical students’ backgrounds and interests may align more with experimental programs, making them more inclined to choose and participate in these types of research. Also, Chinese hospitals and research organizations often pay more attention to medical students’ laboratory experience and achievements when considering different candidates, leading them to focus more on research related to experimental programs to enhance related skills and competitiveness in the job market.

Epidemiological research (26.60%) and entrepreneurship programs (9.57%) may need to be adequately promoted and trained in medical education, resulting in medical students’ less knowledge and interest in these fields.

The situation may vary depending on the school, mentors, and individual student interests and backgrounds. For medical students, balancing participation in different research programs and improving epidemiological research and entrepreneurship skills can give them more opportunities and choices for comprehensive and future career development.

#### Clinical research competencies

For the students who took part in epidemiological research, this study adopted CRAI-12 [17], a shortened version of the Clinical Research Appraisal Inventory, to assess the confidence of students in performing clinical research based on self-efficacy theory [19] and social cognitive career theory [20]. The result is shown in Table 2.

**Table 2:** Participant ratings on clinical research competencies

Question	Average score/Number	Frequency
**The I&E program enhance your ability to design the best data analysis strategy for your study**	3.9	
Strong disagree	0	0.00%
Disagree	2	4.00%
Neutral	14	28.00%
Agree	21	42.00%
Strong agree	13	26.00%
**The I&E program enhance your ability to determine an adequate number of subjects for your research program **	3.7	
Strong disagree	0	0.00%
Disagree	5	10.00%
Neutral	14	28.00%
Agree	22	44.00%
Strong agree	9	18.00%
**The I&E program enhance your ability to write the results section of a research paper that clearly summarizes and describes the results, free of interpretative comments**	3.88	
Strong disagree	1	2.00%
Disagree	1	2.00%
Neutral	11	22.00%
Agree	27	54.00%
Strong agree	10	20.00%
**The I&E program enhance your ability to write a discussion section for a research paper that articulates the importance of your findings relative to other studies in the field **	3.66	
Strong disagree	0	0.00%
Disagree	3	6.00%
Neutral	14	28.00%
Agree	20	40.00%
Strong agree	11	22.00%
**The I&E program enhance your ability to select a suitable topic area for study**	3.8	7.60%
Strong disagree	0	0.00%
Disagree	2	4.00%
Neutral	15	30.00%
Agree	24	48.00%
Strong agree	9	18.00%
**The I&E program enhance your ability to dentify faculty collaborators from within and outside the discipline who can offer guidance to the program**	3.78	
Strong disagree	1	2.00%
Disagree	3	6.00%
Neutral	14	28.00%
Agree	20	40.00%
Strong agree	12	24.00%
**The I&E program enhance your ability to set expectations and communicate them to program staff**	3.76	
Strong disagree	0	0.00%
Disagree	5	10.00%
Neutral	10	20.00%
Agree	27	54.00%
Strong agree	8	16.00%
**The I&E program enhance your ability to ask staff to leave the program team when necessary**	3.68	
Strong disagree	2	4.00%
Disagree	2	4.00%
Neutral	15	30.00%
Agree	22	44.00%
Strong agree	9	18.00%
**The I&E program enhance your ability to describe ethical concerns with the use of placebos in clinical research**	3.66	7.32%
Strong disagree	2	4.00%
Disagree	1	2.00%
Neutral	16	32.00%
Agree	24	48.00%
Strong agree	7	14.00%
**The I&E program enhance your ability to apply the appropriate process for obtaining informed consent from research subjects**	3.9	
Strong disagree	1	2.00%
Disagree	2	4.00%
Neutral	11	22.00%
Agree	23	46.00%
Strong agree	13	26.00%

Given that most students are not experiment leaders or only partially involved in the core steps of the program, the Funding factor in CRAI-12 is not considered.

For most questions, students’ responses are between “Agree” and “Strong agree,” indicating that most students believe the program enhances their abilities in the relevant field.

Students often need more confidence regarding questions involving ethics and other related aspects. This may be because students, not being the leaders of the experiment, require a certain level of expertise and understanding in ethics. They must also gain experience managing human resources and working as a team.

#### Experimental research competencies

For the students who primarily participate in the experimental program, a questionnaire based on AMEE Guide No. 69 [18] and an assessment from Kansas City University [16] is used to assess their research competencies. The result is shown in Table 3.

**Table 3:** Participant ratings on experimental research competencies

Question	Average score/Number	Frequency
**The I&E program enhance your ability to raise question**	3.92	
Strong disagree	1	0.83%
Disagree	5	4.17%
Neutral	25	20.83%
Agree	61	50.83%
Strong agree	28	23.33%
**The I&E program sparks your curiosity in a specific field and strengthens your self-learning and self-driven abilities.**	3.98	
Strong disagree	1	0.83%
Disagree	1	0.83%
Neutral	28	23.33%
Agree	60	50.00%
Strong agree	30	25.00%
**The I&E program helps to improve your critical thinking skills.**	3.93	
Strong disagree	1	0.83%
Disagree	4	3.33%
Neutral	24	20.00%
Agree	64	53.33%
Strong agree	27	22.50%
**The I&E program helps you understand the importance and significance of ethics and industry standards.**	3.74	
Strong disagree	1	0.83%
Disagree	10	8.33%
Neutral	30	25.00%
Agree	57	47.50%
Strong agree	22	18.33%
**The I&E program enhances your academic literacy, including academic ethics and awareness of intellectual property rights.**	3.88	3.24%
Strong disagree	2	1.67%
Disagree	4	3.33%
Neutral	24	20.00%
Agree	66	55.00%
Strong agree	24	20.00%
**The I&E program enhances your ability to search for and read professional literature.**	4.02	
Strong disagree	1	0.83%
Disagree	5	4.17%
Neutral	24	20.00%
Agree	51	42.50%
Strong agree	39	32.50%
**The I&E program helps you understand how to use literature evidence to prove your hypothesis.**	4.1	
Strong disagree	1	0.83%
Disagree	2	1.67%
Neutral	19	15.83%
Agree	60	50.00%
Strong agree	38	31.67%
**The I&E program enhances your ability to assess the strengths and limitations of a program.**	3.88	
Strong disagree	1	0.83%
Disagree	1	0.83%
Neutral	34	28.33%
Agree	59	49.17%
Strong agree	25	20.83%
**The I&E program helps you understand how to design and improve experimental procedures or methods based on specific conditions.**	3.90	
Strong disagree	1	0.83%
Disagree	4	3.33%
Neutral	29	24.17%
Agree	58	48.33%
Strong agree	28	23.33%
**The I&E program helps you understand and identify errors and mistakes in a program, and enables you to avoid or correct them through appropriate means.**	4.00	
Strong disagree	1	0.83%
Disagree	2	1.67%
Neutral	24	20.00%
Agree	62	51.67%
Strong agree	31	25.83%
**The I&E program helps you master various practical details, including process details.**	3.90	
Strong disagree	1	0.83%
Disagree	8	6.67%
Neutral	22	18.33%
Agree	60	50.00%
Strong agree	29	24.17%
**The I&E program helps you master experimental methods or other skills.**	3.98	
Strong disagree	2	1.67%
Disagree	2	1.67%
Neutral	22	18.33%
Agree	64	53.33%
Strong agree	30	25.00%

Several factors are considered in the questionnaire, including curiosity, professionalism, program understanding, experimental techniques, analytical ability, and interpersonal and communication skills.

Students’ responses are concentrated between “Agree” and “Strong agree,” indicating that most students believe the program enhances their abilities in the relevant field.

Students exhibit higher confidence in raising questions, sparking curiosity, enhancing self-learning, improving critical thinking, searching for professional literature, and using literature evidence, likely because these skills are closely aligned with their academic and career development and have been adequately taught and practiced. However, students need more confidence in understanding the importance of ethics and industry standards and assessing program strengths and limitations. This may be due to insufficient course content, less effective teaching methods, or individual disinterest. Enhancing ethics and industry standards education and providing more comprehensive program assessment training could help boost student confidence.

#### Entrepreneurship

Out of 188 students, only 18 students actively participated in the entrepreneurship program. Most believe participating in the Innovation and Entrepreneurship Program helps improve their entrepreneurial skills, but they need more confidence in leadership (Table 4).

**Table 4:** Participant ratings on entrepreneurship

Question	Average score/Number	Frequency
**The I&E program improve your problem-solving skills **	3.94	
Strong disagree	0	0.00%
Disagree	0	0.00%
Neutral	4	22.22%
Agree	11	61.11%
Strong agree	3	16.67%
**The I&E program enhance your ability to manage finances**	3.89	
Strong disagree	0	0.00%
Disagree	0	0.00%
Neutral	6	33.33%
Agree	8	44.44%
Strong agree	4	22.22%
**The I&E program enhance your creativity**	3.83	
Strong disagree	0	0.00%
Disagree	0	0.00%
Neutral	5	27.78%
Agree	11	61.11%
Strong agree	2	11.11%
**The I&E program enhance your ability toto persuade others to agree with you.**	3.94	
Strong disagree	0	0.00%
Disagree	0	0.00%
Neutral	4	22.22%
Agree	11	61.11%
Strong agree	3	16.67%
**The I&E program enhance your leadership skills.**	3.72	
Strong disagree	0	0.00%
Disagree	0	0.00%
Neutral	7	38.89%
Agree	9	50.00%
Strong agree	2	11.11%
**The I&E program gives you the confidence to make good decisions.**	3.89	
Strong disagree	0	0.00%
Disagree	0	0.00%
Neutral	4	22.22%
Agree	12	66.67%
Strong agree	2	11.11%

Medicine is highly specialized, and medical students must dedicate significant time to acquiring medical knowledge and skills. This may limit their interest in entrepreneurship. They may be more inclined to focus on clinical practice and medical research, while medical schools must provide sufficient entrepreneurship education and support.

### Data analysis ability

Data is crucial in most life science research, and data analysis skills are indispensable in enhancing the research abilities of medical students. Analytic skills enable medical students to design research studies that are more robust and scientifically rigorous. They can identify appropriate variables to measure, select suitable sample sizes, and determine the most influential research methods. It allows medical students to interpret and analyze research findings effectively, identify potential errors or biases in their research, and proceed with their study under the guidance of data analysis. Furthermore, a deep understanding of data means students can critically evaluate existing research, which helps them to understand the importance of evidence. These abilities are of great significance to students’ academic and career development. The assessment result is shown in Table 5.

**Table 5:** Participant ratings on data analysis ability

Question	Average score/Number	Fequency
**The I&E program helps you realize the importance of data reproducibility and consistency.**	4.02	
Strong disagree	1	0.53%
Disagree	3	1.60%
Neutral	37	19.68%
Agree	98	52.13%
Strong agree	49	26.06%
**The I&E program enhances your ability to select appropriate statistical measures.**	3.91	
Strong disagree	1	0.53%
Disagree	4	2.13%
Neutral	49	26.06%
Agree	90	47.87%
Strong agree	44	23.40%
**The I&E program enhance your ability to analyze and interpret program results according to the data correctly**	4.06	2.16%
Strong disagree	1	0.53%
Disagree	4	2.13%
Neutral	31	16.49%
Agree	99	52.66%
Strong agree	53	28.19%
**The I&E program helps you understand how to proceed with subsequent processes under the guidance of preliminary results.**	3.94	
Strong disagree	1	0.53%
Disagree	6	3.19%
Neutral	41	21.81%
Agree	96	51.06%
Strong agree	44	23.40%

The innovation and entrepreneurship program positively impacts students’ data analysis and problem-solving abilities. Through the program, students can recognize the importance of data, enhance their ability to select appropriate statistical measures, accurately analyze and interpret program results, and proceed with subsequent processes under the guidance of preliminary results.

### Teamwork and communication

Teamwork and communication skills are crucial for developing research abilities in medical students today [14]. The assessment result is shown in Table 6.

**Table 6:** Participant ratings on team-work and communication

Question	Average score/Number	Frequency
**The I&E program enhances your ability to express opinions and information clearly, as well as to listen and understand others.**	3.98	
Strong disagree	2	1.06%
Disagree	4	2.13%
Neutral	38	20.21%
Agree	95	50.53%
Strong agree	49	26.06%
**The I&E program enhances your ability to engage in academic communication, including academic writing and conference exchanges.**	4.02	
Strong disagree	1	0.53%
Disagree	4	2.13%
Neutral	38	20.21%
Agree	92	48.94%
Strong agree	53	28.19%
**The I&E program promotes collaboration with other disciplinary fields, enhancing your ability to apply diverse skills and knowledge from different domains to solve complex problems.**	3.84	2.04%
Strong disagree	3	1.60%
Disagree	11	5.85%
Neutral	41	21.81%
Agree	92	48.94%
Strong agree	41	21.81%
**The I&E program enhances your awareness of the importance of team members and strengthens your ability to manage your own emotions and handle the emotions of others.**	3.95	
Strong disagree	2	1.06%
Disagree	9	4.79%
Neutral	35	18.62%
Agree	93	49.47%
Strong agree	49	26.06%
**The I&E program enhances your ability to identify the strengths of team members.**	3.95	
Strong disagree	2	1.06%
Disagree	6	3.19%
Neutral	41	21.81%
Agree	89	47.34%
Strong agree	50	26.60%
**The I&E program enhances your ability to plan, analyze, and make decisions within a team.**	3.99	
Strong disagree	1	0.53%
Disagree	4	2.13%
Neutral	37	19.68%
Agree	99	52.66%
Strong agree	47	25.00%

Collaborating with professionals from different disciplines, such as doctors, researchers, and laboratory technicians, allows for sharing knowledge and experiences, complementing each other’s expertise, and ultimately improving the quality and efficiency of research programs. Effective communication is essential throughout the research process. Medical students must communicate effectively with team members, clarify research objectives, delegate tasks, and share progress and results, among other things. Strong communication skills facilitate collaboration, enhance team cohesion, and improve efficiency. Whether in future medical practice or medical research, medical students require effective teamwork and communication skills to learn and work efficiently. The Innovation and Entrepreneurship Training Program typically involves 3–5 students working under the guidance of 1–2 mentors, providing them with a small-scale team collaboration opportunity. These programs aim to create a supportive and inclusive environment where students can learn from each other, exchange ideas, and enhance their problem-solving abilities through practical experience.

Most students agree that participation in innovation programs develops teamwork and communication skills. They become better listeners, contributors, and leaders in team collaborations, and their communication skills with other team members and academic professionals are enhanced. However, it is worth noting that there are still disciplinary limitations in innovation programs, and there should be an emphasis on increasing the diversity of disciplines in future programs.

**Fig. 3:**
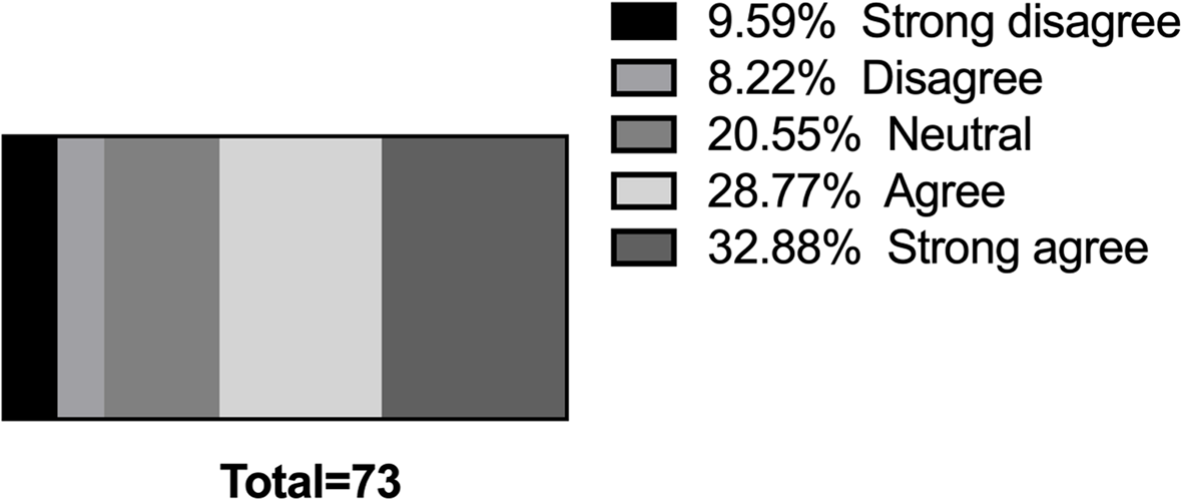
Agreement Levels among Program Leaders Regarding Task Distribution in the I&E Program

To investigate potential issues related to teamwork in the I&E Program, one item in the survey was designed to inquire whether the program leader took on the majority of tasks, thereby limiting the active participation of other team members. 62.5% agreed or strongly agreed that they have taken on most of the program work without assigning tasks for other members to be deeply involved in the program (Fig. 3). This suggests that the operation of most innovation programs may be satisfactory with teamwork.

### Satisfaction and improvement

On the positive side, 73.93% of students are satisfied with the innovation and entrepreneurship programs. 77.12% of the students agreed that the I&E Program positively impacts their research abilities (Table 7).

**Table 7:** Participant satisfaction and improvement ratings for the I&E program

Question	Average score/Number	Frequency
**You are generally satisfied with the I&E program**	3.90	
Strong disagree	2	1.06%
Disagree	10	5.32%
Neutral	37	19.68%
Agree	95	50.53%
Strong agree	44	23.40%
**Your program is strong in theory, but weak in practical implementation**	3.48	
Strong disagree	7	3.72%
Disagree	24	12.77%
Neutral	57	30.32%
Agree	71	37.77%
Strong agree	29	15.43%
**Your I&E program did not achieve the expected results due to insufficient support in objective resources (funding, equipment, etc.)**	3.54	
Strong disagree	4	2.13%
Disagree	25	13.30%
Neutral	57	30.32%
Agree	70	37.23%
Strong agree	32	17.02%
**The undergraduate compulsory courses are affected by the I&E program**	3.01	
Strong disagree	17	9.04%
Disagree	48	25.53%
Neutral	56	29.79%
Agree	51	27.13%
Strong agree	16	8.51%
**Curriculums benefit from the I&E program**	3.66	
Strong disagree	3	1.60%
Disagree	14	7.45%
Neutral	56	29.79%
Agree	86	45.74%
Strong agree	29	15.43%
**Participation in innovation and entrepreneurship programs has a positive impact on the research abilities for medical students.**	3.98	
Strong disagree	1	0.53%
Disagree	5	2.66%
Neutral	37	19.68%
Agree	98	52.13%
Strong agree	47	25.00%
**You may experience negative emotions and even have thoughts of giving up due to the I&E program**	3.08	
Strong disagree	15	7.98%
Disagree	44	23.40%
Neutral	64	34.04%
Agree	41	21.81%
Strong agree	24	12.77%

Although students have different opinions on whether the I&E Program affected their curriculum learning, 61.17% of students believe participating in the program helps their learning. Students with good time management and self-discipline can often effectively balance their research and course learning. Therefore, they tend to believe that the program did not affect their compulsory course learning and didn’t experience negative emotions related to the programs.

On the other hand, students who lack these skills may need more energy from the program, resulting in insufficient focus on their compulsory courses and thoughts of giving up. Other factors also influence this viewpoint. Some curriculums aim to develop students’ research abilities and even require them to participate in innovation and entrepreneurship programs. Students may invest unnecessary energy if the program is too complex, needs more smooth teamwork, or needs more guidance.

On the negative side, 53.19% of students believe their programs are vital in theory but weak in practical implementation. This is due to various reasons, such as insufficient funds, limited resources, lack of guidance, and insufficient long-term follow-up. Similarly, 54.25% of students agreed that their programs had yet to achieve the expected results due to a lack of support (Table 7).

To understand the purpose of medical students participating in innovation and entrepreneurship programs, a multiple-choice question was designed to inquire about the objectives of their involvement.

**Fig. 4:**
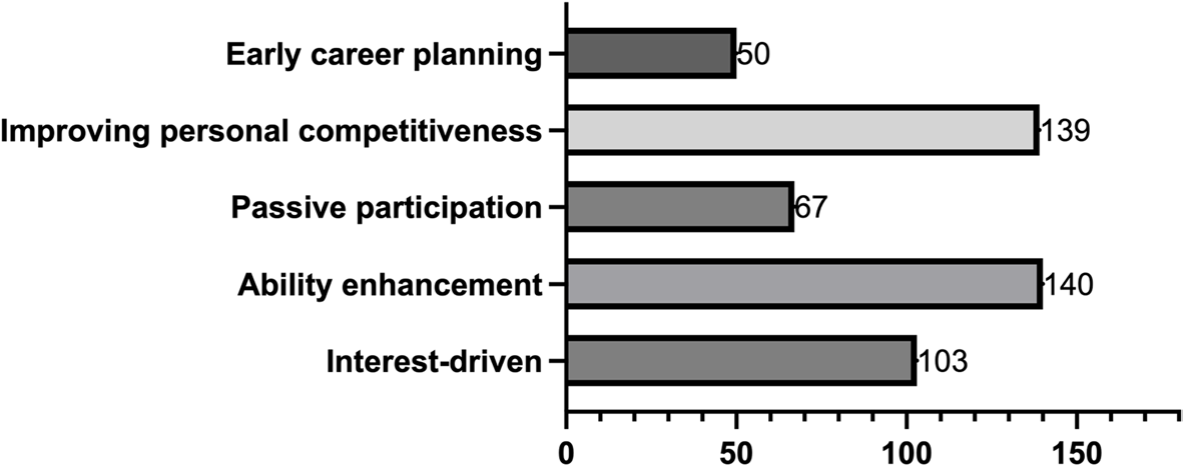
Student Objectives for Participating in the I&E Program

Most students participate in the program to enhance their abilities and improve personal competitiveness, as they recognize the importance of research skills in their medical careers during their undergraduate studies. Only 54.78% of students are driven by interest as they engage in the innovation and entrepreneurship program due to their curiosity about scientific questions. 35.64% of students passively participate in the program, either following the crowd or under the request of their teachers. 26.60% of students agree that they aim to engage in early career planning. The innovation and entrepreneurship program is crucial for the career planning of medical undergraduates, as most of them will pursue a master’s degree after graduation. This program helps them gain early exposure to research and determine whether they aspire to become scientists in the future (Fig. 4).

**Fig. 5:**
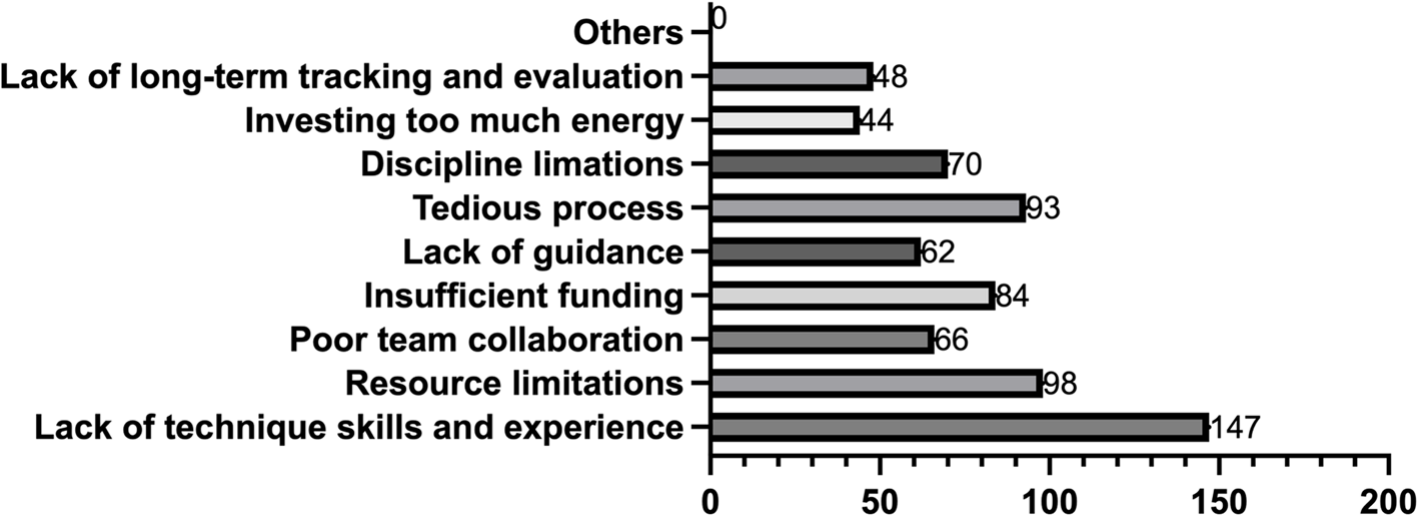
Challenges Faced by Students in the I&E Program

Since approximately half of the students believed that their program did not achieve the expected results, a multiple-choice question was designed to investigate the difficulties encountered by the students. The result is shown in Fig. 5. Among them, 78.19% of students faced problems due to a lack of technical skills and experience, which is reasonable because most students were initially exposed to scientific research through innovation and entrepreneurship programs. 52.12%, 49.46%, and 44.69% of students, respectively, stated that they encountered difficulties due to resource limitations, tedious processes, and insufficient funding. Suggested improvement methods include providing more support regarding resources or funding and improving the standard operating procedure, such as enhancing the application procedure and fund reimbursement.

37.23% of students experienced difficulties due to disciplinary restrictions, while 32.97% and 35.10% encountered problems with their mentors and team members. Measures such as promoting the mentor’s attention to the program and improving the collaborative mode of innovation and entrepreneurship programs may be necessary. Some students (23.40%) felt overwhelmed due to excessive involvement. 25.53% of students needed long-term tracking and evaluation of their programs, which is crucial as it ensures the proper utilization of resources and funding and encourages students to execute their programs consistently.

**Fig. 6:**
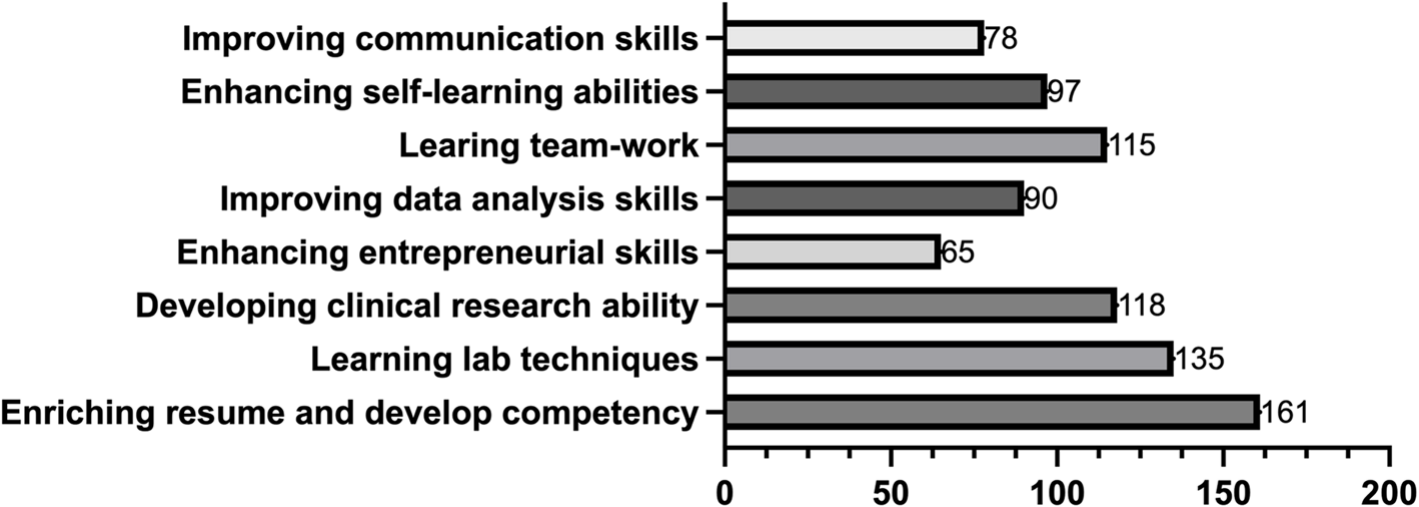
Skills Enhancement Perceived by Students in the I&E Program

**Fig. 7:**
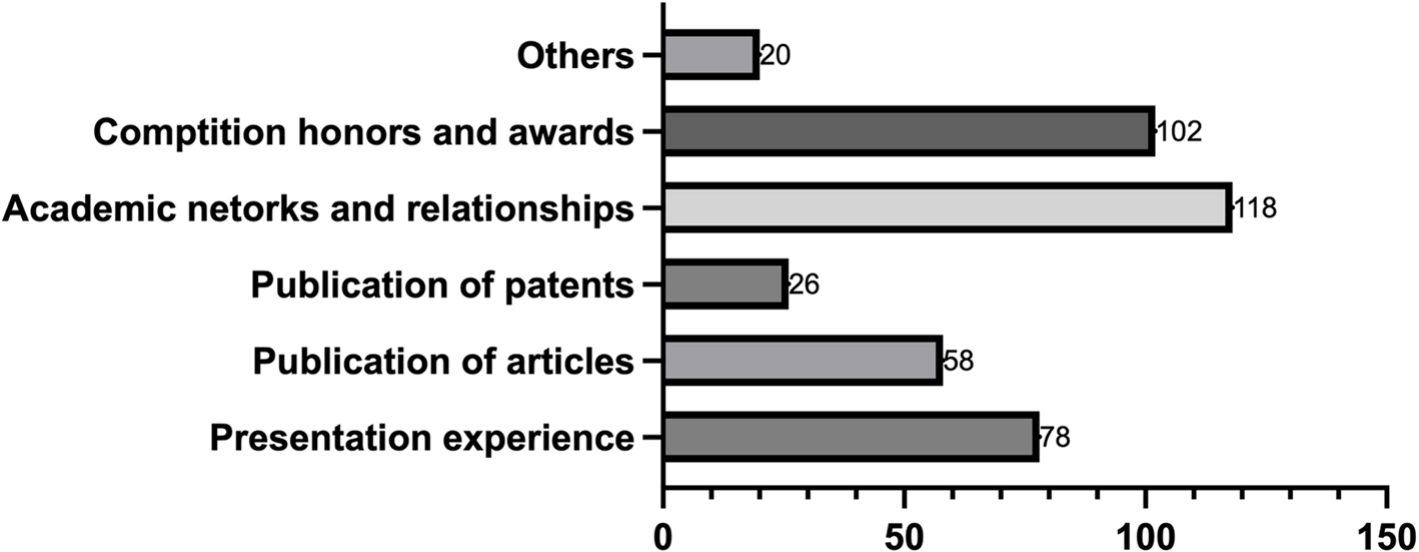
Achievements and Honors Attained by Students in the I&E Program

Two multiple-choice questions are asked from the perspectives of subjective ability enhancement (Fig. 6) and objective awards and honors (Fig. 7) to inquire about the gains that students have obtained from the innovation and entrepreneurship program.

Most students agree that participating in innovation and entrepreneurship programs can enrich their resume and develop their career competency. This practical perspective is widely accepted among students. More than half of the students agree that medical students participate in innovation and entrepreneurship programs to develop laboratory techniques and clinical research abilities and learn teamwork and self-learning skills. Less than half of the students intend to improve their communication and data analysis abilities, with only 34.57% expressing a desire to enhance their entrepreneurial skills (Fig. 6).

The I&E Program has been highly beneficial for medical students. 54.26% of students gained competition awards and honors through their experience in the program. 62.77% of students acknowledged that their network was improved, which would benefit their future careers. 30.85% and 13.83% of students directly or indirectly obtained articles or patent publications due to the program. 41.49% of students have gained public speaking experience, which could be their first academic presentation, contributing to their development as capable and confident researchers (Fig. 7).

## Discussion

Historically, I&E education has focused on business [21], but it’s now expanding into medical education due to evolving healthcare demands [22]. We indicate the necessity of emphasizing the importance of the I&E program in China. Our survey targets students, but the primary obstacle to promoting this medicine program is the need for teachers to become familiar with I & E concepts, resulting in a shortage of new teaching methods [9]. Solutions to this issue include enhancing interdisciplinary collaboration between schools such as design, engineering, and architecture faculties and continuous faculty training to foster understanding of entrepreneurial/innovative concepts.

I&E education is key to boosting the employability of medical students [4]. Our study shows that students enhance teamwork, communication, and clinical research skills, aiming to increase competitiveness through these programs. They achieve awards, expand academic networks, and publish articles or patents. What’s more, the program helps educators link interests to future careers, enhancing students’ satisfaction with career planning.

According to surveys conducted in many countries, incorporating research as a part of undergraduate medical education is considered a potential approach to lay the foundation for future physician-scientists [23, 24]. This may be the direction for developing the I&E Program in China. Students typically view their experiences in medical school research as favorable, often finding them stimulating in generating interest in research and cultivating scholarly research skills [25]. In our study, medical students who have participated in the I&E Program not only objectively acquire a variety of laboratory or theoretical research techniques and methods but also subjectively gain confidence in their potential to become future scientists, entrepreneurs, and collaborative team members based on the Self-Efficacy Theory [19] and Social Cognitive Career Theory [20]. The theory of self-efficacy states that believing in your ability to do something makes you more likely to succeed at it. Social cognitive career theory connects the anticipation of favorable results and achievements in specific behaviors to the inclination to pursue a career that demands these behaviors. Confidence doesn’t always mean ability, but being more confident and interested in clinical research shows that research training is helpful [26].

Undergraduate research is essential for progress in clinical medicine. Early research training linked to health practices helps tackle global health challenges effectively [27]. Surveys and our research indicate that undergraduate medical students generally hold a favorable view of scientific research [28, 29]. However, the number of research students still needs to be higher [30]; they encounter several obstacles that hinder their engagement and contribution to the initiation and advancement of scientific projects. They need to balance their projects with their studies while investing more time and interacting with their tutors. It is worth noting that instruction from teachers is important in accomplishing the project [31]. On the other hand, students complain about inadequate funding. Approximately half of the students feel their programs need more support during implementation and fail to achieve the expected outcomes.

Contrary to what one might expect, most students do not believe the I&E Program will impact their mandatory coursework. More than half of the students believe that participating in the I&E Program contributes to their learning in required courses. This could be attributed to the fact that I&E education is woven into numerous undergraduate mandatory compulsory courses, and students recognize the significance of their current career planning. According to a previous study, there is a substantial overlap in crucial attributes from both professional and research standpoints [5], indicating a close integration between that research and the curriculum.

Most students expressed satisfaction with the programs. Students’ enjoyment contributes to enhancing their learning outcomes [32]. However, it’s noteworthy that many students have experienced negative emotions during the I&E Program and have even contemplated giving up. Further investigation is needed to uncover the specific reasons behind these experiences. What can be confirmed is that some students noted a disconnect between theoretical knowledge and practical implementation. Challenges included technical skill gaps, resource limitations, mentor issues, and team dynamics. Students highlighted the need for improved support, resources, and streamlined procedures.

According to the General Medical Council’s Tomorrow Doctor, teamwork is an important learning goal in medical curricula. Efficient health systems feature continuity, physician-patient partnerships, teamwork among healthcare providers, and inter-facility communication [33]. However, 35.10% of students reported teamwork difficulties, and 62.5% of program leaders acknowledge that they have taken on most of the work in the program, implying that other team members may have made minimal contributions and are likely to have yet to gain any skills. In such a situation, the data they provide may deviate from the actual impact of the I&E Program. To address these challenges, the I&E Program may benefit from implementing structured team-building exercises and fostering a culture of open communication and mutual respect among participants.

Based on teamwork, interdisciplinary collaboration is also essential for medical students as it improves their clinical skills and equips them with the ability to navigate the complexities of modern healthcare, foster innovation, and provide high-quality patient-centered care [34]. Considering students’ epistemic backgrounds, individual learning preferences, and the potential for synergy is crucial when designing interdisciplinary educational courses for a meaningful and effective learning experience [35]. As previously mentioned, multidisciplinary collaboration between medical schools and other schools also helps to improve I&E pedagogy.

When asking students for suggestions for improvement regarding the I&E Program, the most common complaints revolved around insufficient funding, often requiring them to self-finance or seek instructor assistance. In 2023, Central South University reduced its support for medical innovation projects. This reduction is evident as the number of projects receiving provincial or national-level support dropped to 173, a significant decline compared to 353 in 2022 and 291 in 2021. Additionally, there has been a notable increase in projects receiving less financial support, with most projects (56%) not receiving any financial backing [3]. Even the highest level of funding support is considered insufficient (about 10,000 ¥).

There needs to be more interest in research careers among medical students upon graduation in the US [36]. This is accompanied by a declining number of MDs seeking support from the NIH loan repayment program, a growing age at which physicians achieve their first R01 grant success, and fewer physicians considering research as their primary work activity [37]. In China, a similar situation of decreased or stagnant research funding is also occurring. The number of programs funded by the National Natural Science Foundation Of China (NSFC) in 2023 has reached 48,785, an increase of 468 compared to 2022. However, amidst a significant rise in the total number of program applications, the funding ratio continues to show a declining trend [38]. These trends raise concerns about the stability of the research career path. Furthermore, 2023 also witnessed the NSFC’s initiative to start providing research funding to undergraduate students. Considering these challenges, one recommendation for improvement is to enhance support for medical innovation programs, especially for undergraduate students, to encourage more aspiring physician-scientists.

The I&E Program could benefit from more innovation, as medical students often struggle to select research topics on their own. Improving the program may involve a budget increase and adopting active, interdisciplinary teaching methods to better engage students in research and provide more support. In conclusion, overcoming challenges with more support and mentorship prepares students for future careers through research and interdisciplinary education.

## Limitation

Our questionnaire survey aimed to understand the basic situation of innovative education in medical schools in our country. The cross-sectional design and lack of a control group are limitations of this study. The online questionnaire distribution may lead to subjective and recall biases. Due to inconsistencies in concepts, as well as limitations in the development of the questionnaire, we identified specific competencies to assess students’ acquisition of I&E competencies from the I&E Program. However, this may be partial and could be improved upon.

## Data Availability

All data generated or analyzed during this study are included in this published article.

## References

[1] Ministry of Education. Proposals on promoting education for innovation and entrepreneurship in higher education and on undergraduate independent entrepreneurship. http://www.moe.gov.cn/srcsite/A08/s5672/201005/t20100513_120174.html. Assessed 15 Mar 2024.

[2] National undergraduate innovation and entrepreneurship. http://gjcxcy.bjtu.edu.cn/Index.aspx. Assessed 20 Oct 2023.

[3] Central South University. https://www.csu.edu.cn/. Assessed 20 Oct 2023.

[4] Niccum BA, Sarker A, Wolf SJ, Trowbridge MJ (2017). Innovation and entrepreneurship programs in US medical education: a landscape review and thematic analysis. Med Educ Online.

[5] Yu M, Liu W (2022). The importance of College students’ innovative entrepreneurial training plan program in cultivation of medical undergraduates’ scientific research literacy. Open J Soc Sci.

[6] Grailer JG, Alhallak K, Antes AL, Kinch MS, Woods L, Toker E, Garbutt JM (2022). A novel innovation and entrepreneurship (I&E) training program for biomedical research trainees. Acad Med.

[7] Brinton TJ, Kurihara CQ, Camarillo DB, Pietzsch JB, Gorodsky J, Zenios SA (2013). Outcomes from a postgraduate biomedical technology innovation training program: the first 12 years of Stanford biodesign. Ann Biomed Eng.

[8] Bacigalupo MKP, Punie Y, Van den Brande G. EntreComp: the entrepreneurship competence framework. Luxembourg: Publication Office of the European Union; 2016. Report No.: EUR 27939 EN.

[9] Suryavanshi T, Lambert S, Lal S, Chin A, Chan TM (2020). Entrepreneurship and innovation in health sciences education: a scoping review. Med Sci Educ.

[10] Audretsch DB, Falck O, Heblich S (2011). Handbook of research on innovation and entrepreneurship.

[11] Garbutt J, Antes A, Mozersky J, Pearson J, Grailer J, Toker E, DuBois J (2019). Validating curricular competencies in innovation and entrepreneurship for biomedical research trainees: a modified Delphi approach. J Clin Transl Sci.

[12] Oecd. Skills for Innovation and research. Paris: OECD; 2011.

[13] Valenta AL, Meagher EA, Tachinardi U, Starren J (2016). Core informatics competencies for clinical and translational scientists: what do our customers and collaborators need to know?. J Am Med Inform Assoc.

[14] Chandrashekar A, Mohan J (2019). Preparing for the National Health Service: the importance of teamwork training in the United Kingdom medical school curriculum. Adv Med Educ Pract.

[15] Parry SB (1996). Just what is a competency?(And why should you care?). Training..

[16] Adkison LR, Glaros AG (2012). Assessing research competency in a medical school environment. Med Sci Educ.

[17] Robinson GFWB, Switzer GE, Cohen ED, Primack BA, Kapoor WN, Seltzer DL (2013). A shortened version of the clinical research appraisal inventory. Acad Med.

[18] Laidlaw A, Aiton J, Struthers J, Guild S (2012). Developing research skills in medical students: AMEE Guide No. 69. Med Teach.

[19] Bandura A (1977). Self-efficacy: toward a unifying theory of behavioral change. Psychol Rev.

[20] Lent RW, Brown SD, Hackett G (1994). Toward a unifying social cognitive theory of career and academic interest, choice, and performance. J Vocat Behav.

[21] Kuratko DF (2005). The emergence of entrepreneurship education: development, trends, and challenges. Entrep Theory Pract.

[22] Arshad S, Huda NU, Nadeem N, Ali S, Ahmad N, Anwar S (2021). Perceptions of medical students about research at undergraduate level. J Ayub Med Coll Abbottabad.

[23] Laskowitz DT, Drucker RP, Parsonnet J, Cross PC, Gesundheit N (2010). Engaging students in dedicated research and scholarship during medical school: the long-term experiences at Duke and Stanford. Acad Med.

[24] Mahomed S, Ross A, Wyk J (2021). Training and assessing undergraduate medical students’ research: learning, engagement and experiences of students and staff. Afr J Prim Health Care Fam Med.

[25] Chang Y, Ramnanan CJ (2015). A review of literature on medical students and Scholarly Research. Acad Med.

[26] Mullikin EA, Bakken LL, Betz NE (2007). Assessing research self-efficacy in physician-scientists: the clinical research APPraisal inventory. J Career Assess.

[27] Schexnayder S, Starring H, Fury M, Mora A, Leonardi C, Dasa V (2018). The formation of a medical student research committee and its impact on involvement in departmental research. Med Educ Online.

[28] Hren D, Lukić IK, Marusić A, Vodopivec I, Vujaklija A, Hrabak M, Marusić M (2004). Teaching research methodology in medical schools: students’ attitudes towards and knowledge about science. Med Educ.

[29] Vujaklija A, Hren D, Sambunjak D, Vodopivec I, Ivaniš A, Marušić A, Marušić M (2010). Can teaching research methodology influence students’ attitude toward science? Cohort study and nonrandomized trial in a single medical school. J Investig Med.

[30] Muhandiramge J, Vu T, Wallace MJ, Segelov E (2021). The experiences, attitudes and understanding of research amongst medical students at an Australian medical school. BMC Med Educ.

[31] Murdoch-Eaton D, Drewery S, Elton S, Emmerson C, Marshall M, Smith JA (2010). What do medical students understand by research and research skills? Identifying research opportunities within undergraduate projects. Med Teach.

[32] Blunsdon B, Reed K, McNeil N, McEachern S (2003). Experiential learning in social science theory: an investigation of the relationship between student enjoyment and learning. High Educ Res Dev.

[33] Epstein RM (2002). Defining and assessing professional competence. JAMA.

[34] Spelt EJH, Biemans HJA, Tobi H, Luning PA, Mulder M (2009). Teaching and learning in interdisciplinary higher education: a systematic review. Educ Psychol Rev.

[35] Oudenampsen J, van de Pol M, Blijlevens N, Das E (2023). Interdisciplinary education affects student learning: a focus group study. BMC Med Educ.

[36] Garrison HH, Ley TJ (2022). Physician-scientists in the United States at 2020: trends and concerns. FASEB J.

[37] Jain MK, Cheung VG, Utz PJ, Kobilka BK, Yamada T, Lefkowitz R (2019). Saving the endangered physician-scientist — a plan for accelerating medical breakthroughs. N Engl J Med.

[38] National Natural Science Foundation of China. https://www.nsfc.gov.cn/english/site_1/index.html. Assessed 20 Oct 2023.

